# Content of Carotenoids, Violaxanthin and Neoxanthin in Leaves of *Triticum aestivum* Exposed to Persistent Environmental Pollutants

**DOI:** 10.3390/molecules26154448

**Published:** 2021-07-23

**Authors:** Ocsana Opriş, Florina Copaciu, Maria Loredana Soran, Ülo Niinemets, Lucian Copolovici

**Affiliations:** 1 National Institute for Research and Development of Isotopic and Molecular Technologies, 67-103 Donat, 400293 Cluj-Napoca, Romania; opris.ocsana@yahoo.com (O.O.); loredana.soran@itim-cj.ro (M.L.S.); 2 Department of Chemistry and Biochemistry, University of Agricultural Sciences and Veterinary Medicine, 3-5 Mănăştur, 400372 Cluj-Napoca, Romania; florina_copaciu@yahoo.ro; 3 Institute of Agricultural and Environmental Sciences, Estonian University of Life Sciences, 1 Kreutzwaldi, 51006 Tartu, Estonia; ylo.niinemets@emu.ee; 4 Estonian Academy of Sciences, Kohtu 6, 10130 Tallinn, Estonia; 5 Development and Innovation in Technical and Natural Sciences Institute for Research, Faculty of Food Engineering, Tourism and Environmental Protection, Aurel Vlaicu University of Arad, 2 Elena Drăgoi St., 310330 Arad, Romania

**Keywords:** antibiotics, abiotic stress, carotenoid content, textile dyes, *Triticum aestivum*

## Abstract

Persistent pollutants such as pharmaceuticals, pesticides, musk fragrances, and dyes are frequently detected in different environmental compartments and negatively impact the environment and humans. Understanding the impacts of diffuse environmental pollutants on plants is still limited, especially at realistic environmental concentrations of contaminants. We studied the effects of key representatives of two major classes of environmental pollutants (nine different antibiotics and six different textile dyes) on the leaf carotenoid (violaxanthin and neoxanthin) content in wheat (*Triticum aestivum* L.) using different pollutant concentrations and application times. The wheat plants were watered with solutions of selected environmental pollutants in two different concentrations of 0.5 mg L^−1^ and 1.5 mg L^−1^ for one week (0.5 L) and two weeks (1 L). Both categories of pollutants selected for this study negatively influenced the content of violaxanthin and neoxanthin, whereas the textile dyes represented more severe stress to the wheat plants. The results demonstrate that chronic exposure to common diffusively spread environmental contaminants constitutes significant stress to the plants.

## 1. Introduction

The environmental impacts of persistent organic pollutants have been acknowledged for several years now. Considerable amounts of effluents with organic pollutants are found in different ecological compartments, mainly originating from agricultural runoffs, effluents generated by industries, sewage plants, and many other anthropogenic activities. Many of these organic pollutants found in wastewaters are non-biodegradable. Thus, these pollutants are raising concerns because they are persistent, highly toxic compounds that can transported over long environmental distances and can be bioaccumulated in the tissues of aquatic animals and plants [1,2].

Among the various pharmaceuticals, antibiotics in soil and water ecosystems cause particular concern since their increasing use, and the consequent development of multi-resistant bacteria, pose severe risks for human and animal health [3,4,5]. The fate and behavior of antibiotics in soil have become one of the most complicated problems in environmental chemistry [6]. The physical, chemical and biological properties of sewage sludge have significant importance on the direction of utilization [7].

Also was shown that antibiotic could be uptake by plants or other organisms from soil fertilized with animal manure or wastewaters used for irrigation [8]. Even if the impacts of ingesting antibiotics presenting plants until now are not very well known and investigated, they can cause allergic/toxic reactions and/or increase antibiotic resistance in humans [3]. The adverse effects of antibiotics have recently begun to be investigated [9,10] because the issue of pharmaceuticals in the environment represents a topic of tremendous global interest. The toxicity of antibiotics has been assessed in various living organisms [11], but mostly in algae [12,13,14] because they represent the basis of the food chain in aqueous ecosystems. Also, the effects of antibiotics vary from antibiotic to antibiotic and also vary by plant species. Minden et al. indicated that antibiotics can affect element contents in plants [15]. Chen et al. [16] showed that oxytetracycline exposure diminished the photosynthetic capacity of rape. Margas et al. [17] demonstrated that the physiology and biochemistry of pea seedlings are strongly impacted by soil tetracycline.

The textile industry has a significant impact on the environment. This industry consumes large volumes of water, energy, and chemicals, and the wastewaters from textile processing operations contain many residues, including a large number of dye residues. The textile dyes are highly stable (half-life 2–13 years); they have been detected in many streams and are considered to have genotoxic and cytotoxic effects on the aquatic environment [17]. Regarding the ecotoxicological aspects of the dyes, studies have investigated the impact on microorganisms under aerobic conditions, mutagenicity character of complexes azo dyes containing metal cations (cobalt, chromium, and iron) in *Salmonella* [17] and plants [18]. Plant studies have demonstrated a decrease in the content of carbohydrates, protein, and chlorophyll, indicating a disruption of plant growth [18,19].

Our previous studies [9,10] demonstrated that antibiotics and textile dyes had moderate effects on wheat foliage photosynthetic characteristics. The emission of stress volatiles was the most sensitive indicator of pollutants treatments. Also, it was demonstrated that antibiotics and textile dyes modified the wheat flavonoids content [20]. On the other hand, carotenoids play essential roles in photosynthesis, light-harvesting, prevention of photo-oxidative damage, and serving as precursors for biosynthesis of the phytohormones abscisic acid (ABA) and strigolactone. Carotenoids also quench free radicals and dissipate the excess excitation energy. Thus, the play an essential role in alleviating the oxidative stress caused by severe abiotic stressors, including pollutant stress [21,22]. The determination of violaxanthin and neoxanthin from *Triticum aestivum* L. leaves under stress conditions is a step forward to understanding the xanthophyll cycle involved in the primary photochemical processes of PSII, especially in high light.

The present study on the influence of antibiotics and textile dyes on the carotenoids of wheat leaves complements previous ones. The current study presents the effects of a range of antibiotics and textile dyes on carotenoid (violaxanthin and neoxanthin) contents in wheat plants (*Triticum aestivum* L.). The stress application consisted of watering the wheat plants with solutions of nine different antibiotics and six different textile dyes in two concentrations (0.5 mg L^−1^ and 1.5 mg L^−1^) which were selected to correspond with moderately high environmental concentrations [10,23,24]. The antibiotics chosen for this study were from five different classes: penicillins (amoxicillin, ampicillin, penicillin G; cephalosporins (ceftazidime and ceftriaxone), tetracyclines (tetracycline and doxycycline), a fluoroquinolone (ciprofloxacin), and macrolide (erythromycin). The textile dyes selected were two anthraquinone dyes (Optilan Blue and Lanasyn Blue) and four azo dyes (Lanasyn Red, Nylosan Red, Nylosan Dark Brown, and Lanasyn Dark Brown). We hypothesized that the selected antibiotics and textile dyes would reduce the violaxanthin and neoxanthin contents in wheat in a dose-dependent manner. Chemometric techniques were used as tools for the characterization of treatment differences based on violaxanthin and neoxanthin contents.

## 2. Results

**Figure 1:**
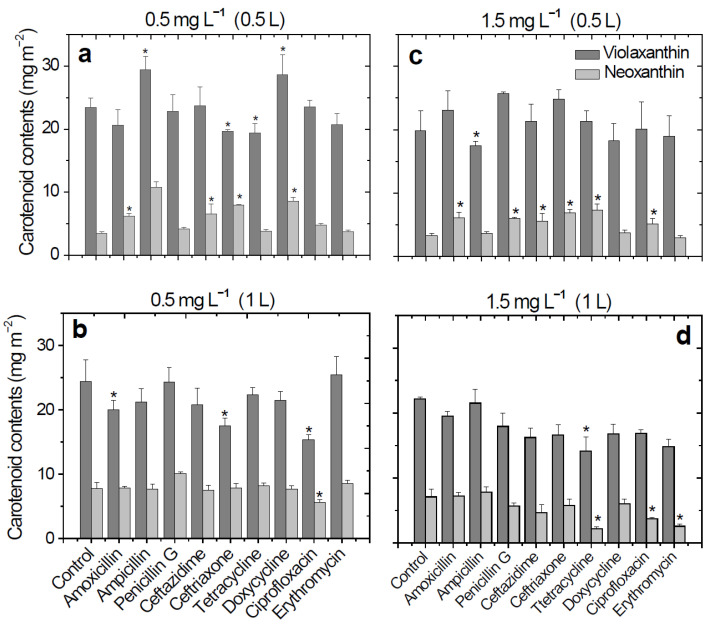
Changes in carotenoid (violaxanthin and neoxanthin) content (mg m^−2^) in *Triticum aestivum* L. cv. “Lovrin” plants in response to treatments with nine antibiotics. The plants were watered with a total volume of antibiotic solutions of either 0.5 L (one-week-old plants) or 1 L (two-week-old plants) at a concentration of either 0.5 mg L^−1^ (**a**,**b**) or 1.5 mg L^−1^ (**c**,**d**). Each data point represents the mean (± SEM) of three independent replicate experiments with a different plant. “*” demonstrates statistically significant differences between the given antibiotic treatment and the control (*P* < 0.05).

Significant decreases in violaxanthin levels were observed, even at an antibiotic solution concentration of 0.5 mg L^−1^ (0.5 L, Figure 1). These decreases were observed in wheat plants that had been treated with antibiotics from the cephalosporin (ceftriaxone, 16.4%) and tetracycline (tetracycline, 18.4%) classes (Figure 1a). In the case of ceftriaxone treatment, a decrease in the content of violaxanthin (28.3%) was observed after the plants were watered with a larger volume of antibiotic solution (1 L, Figure 1b). The lowest violaxanthin content was obtained in the ciprofloxacin antibiotic treatment of 0.5 mg L^−1^, 1 L (15.3 mg m^−2^). For the higher concentration of the antibiotic solutions used for watering the plants (1.5 mg L^−1^), the content of violaxanthin decreased with the volume of the antibiotic solutions used for watering the plants (Figure 1c,d). Using antibiotic concentrations of 1.5 mg L^−1^, 0.5 L, for the plant treatment, the violaxanthin content slightly increased except for ampicillin, doxycycline, and erythromycin (Figure 1c). After using a higher volume (1 L) of the same antibiotic concentration, the content of the violaxanthin decreased in all cases (decreases between 3.0–36.5%). The lowest content of violaxanthin was obtained in the case of tetracycline treatment (14.1 mg m^−2^, Figure 1d).

In the case of neoxanthin, significant decreases were obtained in tetracycline (2.21 mg m^−2^, 68.9%), ciprofloxacin (3.76 mg m^−2^, 47.1%), and erythromycin (2.59 mg m^−2^, 63.6%) treatments (Figure 1d). Overall, neoxanthin and violaxanthin contents decreased with increasing concentrations and volumes of antibiotic solutions used for watering the wheat plants.

**Figure 2:**
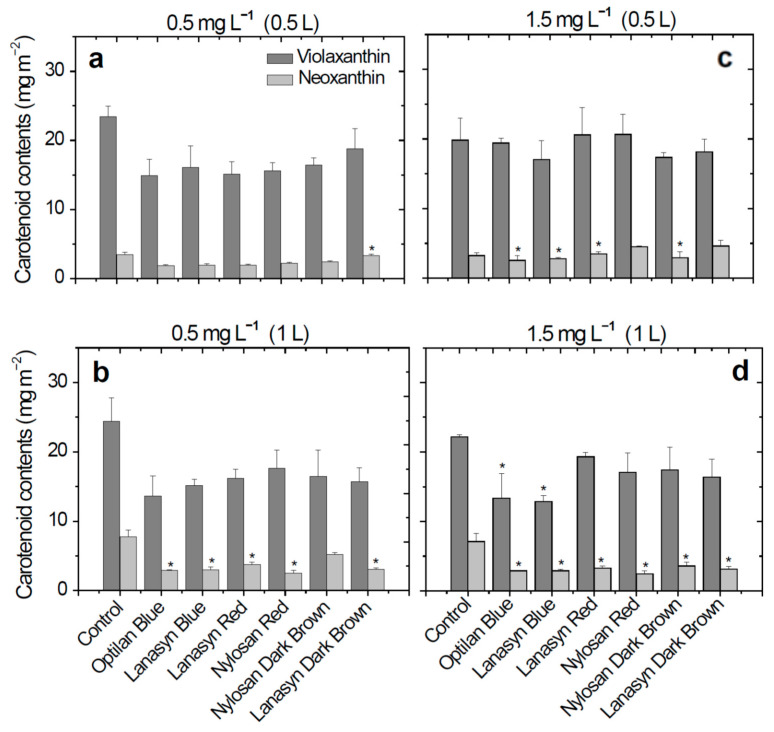
Changes in carotenoid (violaxanthin and neoxanthin) contents (mg m^−2^) in *Triticum aestivum* L. cv. “Lovrin” plants in response to treatments with six textile dyes. The plants were watered with a total volume of textile dye solutions of either 0.5 L (one-week-old plants) or 1 L (two-week-old plants) at a concentration of either 0.5 mg L^−1^ (**a**,**b**) or 1.5 mg L^−1^ (**c**,**d**). “*” demonstrates statistically significant differences between the given antibiotic treatment and the control (*P* < 0.05). The symbols above the columns stand for statistical differences, as in Figure 1.

After the treatment with a volume of 0.5 L solution of textile dyes (1 week) with a concentration of 0.5 mg L^−1^, neoxanthin content slightly decreased in plants treated with textile dyes solution (Figure 2a). Furthermore, after two weeks of treatment (1 L of solution applied) for the same concentration, significant decreases in neoxanthin content were observed in plants treated with solutions of the anthraquinone dyes Optilan Blue (2.89 mg m^−2^, 62.8%) and Lanasyn Blue (2.98 mg m^−2^, 61.5%), and the azo dyes Lanasyn Red (3.71 mg m^−2^, 52.1%), Nylosan Red (2.53 mg m^−2^, 67.3%), and Lanasyn Dark Brown (3.04 mg m^−2^, 60.8%), (Figure 2b).

In the treatment with a concentration of 1.5 mg L^−1^, neoxanthin content was significantly reduced by ome textile dyes in both weeks of treatment (Figure 2c,d). After the first week of treatment (0.5 L) with a concentration of 1.5 mg L^−1^, a decrease in the neoxanthin content was observed for the treatment with the Optilan Blue (2.57 mg m^−2^, 21.38%) and Lanasyn Blue (2.79 mg m^−2^, 14.8%) anthraquinone dyes and the treatment with the azo dye Nylosan Dark Brown (2.94 mg m^−2^, 10.2%), in comparison with the control plants. After two weeks of treatment (1 L) with the same concentration (1.5 mg L^−1^), a decrease in neoxanthin content (49.4–65.5%) was observed in the case of all treatments with textile dyes. (Figure 2d).

In the case of violaxanthin, no significant differences were recorded in the plants treated with textile dyes in the concentration of 0.5 mg L^−1^, 0.5 L, and 1 L (Figure 2a,b) and 1.5 mg L^−1^, 0.5 L (Figure 2c). Significant decreases were observed in the treatments with Optilan Blue (13.33 mg m^−2^, 39.9%), and Lanasyn Blue (12.86 mg m^−2^, 42.04%), anthraquinone textile dye in the concentration of 1.5 mg L^−1^ and the final volume of 1 L (Figure 2d).

**Figure 3:**
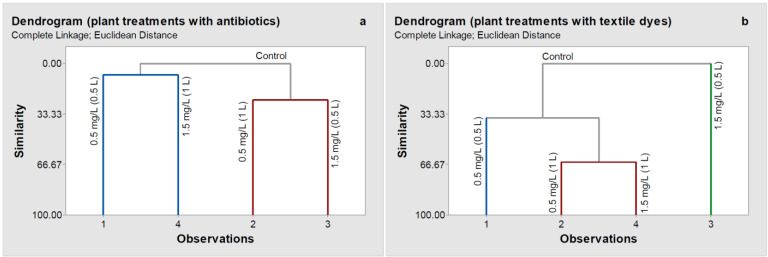
Hierarchical clustering dendrograms for leaf carotenoid contents (violaxanthin and neoxanthin) in wheat treated with antibiotics (**a**) and textile dyes (**b**). The dendrograms were created by the complete linkage method.

In the case of the treatments with antibiotics (Figure 3a), the hierarchical cluster analysis (HCA) based on HPLC chromatograms grouped all the samples into three main clusters: control plants, plants treated with a concentration of 0.5 mg L^−1^ (0.5 L) and 1.5 mg L^−1^ (1 L) antibiotics solution, and plants treated with 0.5 mg L^−1^ (1 L) and 1.5 mg L^−1^ (0.5 L) antibiotics solution. Thus, simultaneous consideration of violaxanthin and neoxanthin further reinforced the different responses of wheat plants to the stress resulting from different antibiotics contents. Compared to the control plants, the most significant changes were obtained in the plants treated with 0.5 mg L^−1^ (1 L) and 1.5 mg L^−1^ (0.5 L) antibiotics solution. The plants reacted by decreasing carotenoid content at the lowest antibiotic concentration used after watering them with 1 L antibiotics solution. In the case of 1.5 mg L^−1^ antibiotics concentration, carotenoid content decreased even after wetting them 0.5 L antibiotics solution.

In the case of the textile dye treatments, HCA analysis grouped the samples in four clusters: control, plants treated with a concentration of 1.5 mg L^−1^ (0.5 L) textile dye solutions, plants treated with a concentration of 0.5 mg L^−1^ (0.5 L) textile dye solutions, and the last one consisted of the plants treated with 0.5 mg L^−1^ (1 L) and 1.5 mg L^−1^ (1 L). Thus, the wheat plants were sensitive even to the lowest concentration of textile dyes, and there was a much clearer dose-dependence than for treatments with antibiotics.

## 3. Discussion

In many studies looking at the effects of environmental pollutants on plants, dose-dependent changes in plant activity have been demonstrated [9,10]. In the current study, we were interested in the effects of environmentally realistic concentrations of antibiotics and textile dyes on wheat plants. Thus, we used pollutant concentrations of 0.5 mg L^−1^ and 1.5 mg L^−1^. These concentrations are lower than in other similar studies [25,26] but correspond to concentrations that have been detected in environmental samples (contaminated rivers, soils, and sewage water). On the other hand, we used chronic exposures for one week (watering with 0.5 L of the solution with given pollutant concentration) and for two weeks (watering with 1 L of solution). Overall, both categories of pollutants selected for this study negatively influenced the content of violaxanthin and neoxanthin (Figure 1 and Figure 2).

In contrast, the textile dyes caused more severe stress to the wheat plants. With the increasing concentration of textile dyes, the content of the selected carotenoids progressively decreased, while in the case of the antibiotics, such a clear dose-dependence was not observed. This can be caused by the fact that textile dyes have chelating metal-binding sites that could reduce the concentrations of essential metal co-factors such as Mg^2+^ needed for the activity of different terpenoid synthesis pathway enzymes [27,28]. On the other hand, both textile dyes and antibiotics directly affect the enzymes of the primary metabolism [29]. Reduction in primary metabolism can enhance the synthesis of carotenoids, thereby alleviating oxidative stress. However, for these two carotenoids, such a positive effect of stress was not observed. Previous work has shown that plant metabolites are affected more by treatments with greater dye concentrations [9,20].

**Figure 4:**
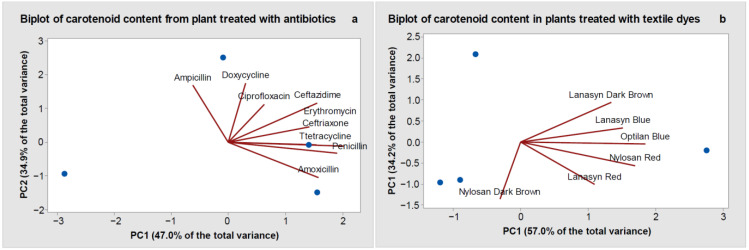
Principal component analysis (biplot representations) based on leaf carotenoid contents (violaxanthin and neoxanthin) in wheat plants treated with antibiotics (**a**) and textile dyes (**b**).

Principal component analyses (PCA) further demonstrated quantitative differences in the effects of different antibiotics (Figure 4a) and textile dyes (Figure 4b) on carotenoid content. In the antibiotic treatments (Figure 4a), PC1 with variance 4.23 (equal to the largest eigenvalue) accounted for 47.0% of the total variation in the data. PC2 (variance 3.14) accounted for 34.9% of the total data variation. PC1 was represented by amoxicillin, penicillin, ceftazidime, ceftriaxone, tetracycline, and erythromycin, while PC2 was represented by ampicillin, doxycycline, and ciprofloxacin (Figure 4a). In textile dye treatments, the eigenvalues were 3.42 for PC1 and 2.05 for PC2, and PC1 accounted for 57% and PC2 for 34.2% of the data variability. The PC1 was represented by Optilan Blue, Lanasyn Blue, Lanasyn Red, Nylosan Red, Lanasyn Dark Brown, and PC2 by Nylosan Dark Brown (Figure 4b).

Plants respond by different adaptive mechanisms to different environmental stresses, including alterations in different sets of plant metabolites [9]. Plant carotenoid composition and content depend particularly strongly on environmental conditions. Because carotenoids play essential roles in the dissipation of energy absorbed in excess and serve as antioxidants [30], plants deficient in carotenoids suffer from photodamage [31]. Thus, the reduction of carotenoid contents in response to antibiotic and textile dye treatments suggests that plants in diffusely polluted environments might be more vulnerable to photoinhibition. Thus, chronic exposure to low-level pollutants can significantly curb plant photosynthetic productivity.

## 4. Materials and Methods

### 4.1. Plant Material and Growing Conditions

Wheat (*Triticum aestivum* L. cv. “Lovrin”, Fundulea, Romania) seeds were sown (depth 1 cm) in commercial garden soil contained in 1 L (10 × 10 × 10 cm) plastic pots that included a slow-release NPK fertilizer with microelements (Biolan, Eura, Finland). A Percival growth chamber (model LT36VL, Percival Scientific, Inc., Perry, IA, USA) was used for growing the wheat plants under environmentally-controlled conditions with a light intensity of 500 µmol m^−2^ s^−1^ provided for 12 h light period, and day/night temperatures set at 25/18 °C.

### 4.2. Stress Application

The experiment started when the coleoptiles had emerged and the second leaf had reached at least 50% of its final length (Zadoks growth stage 1.2; [32]), 14 days after sowing the seeds. The plants were watered every day with aqueous solutions of selected antibiotic and textile dyes in concentrations of either 0.50 mg L^−1^ or 1.50 mg L^−1^. The control plants received equal amounts of distilled water.

The carotenoid extraction and analysis were performed at 7 and 14 days after starting the experiment when the wheat plants had been watered with a total volume of 0.5 L (day 7), 1 L (day 14) of the given antibiotic and dye solutions. All the treatments (nine different antibiotics and six different textile dyes, in two concentrations, and the control) were replicated three times.

The antibiotics, amoxicillin, ampicillin, penicillin G, ceftriaxone, and tetracycline were purchased from Antibiotice (Iaşi, Romania), ceftazidime from GlaxoSmithKline (Braşov, Romania), doxycycline and erythromycin from Sandoz (Târgu-Mureş, Romania), and ciprofloxacin from Ranbaxy (Cluj-Napoca, Romania). All textile dyes (Optilan Blue, Lanasyn Blue, Lanasyn Red, Nylosan Red, Nylosan Dark Brown, and Lanasyn Dark Brown) were purchased from Clariant Produkte (Muttenz, Switzerland).

### 4.3. Carotenoids Extraction and Analysis

Wheat leaf samples (4 cm^2^) were powdered in liquid nitrogen with some calcium carbonate using a mortar and a pestle. The carotenoids were extracted in ice-cold 100% acetone. After centrifuging the extracts at 0 °C and 9500× *g* for 3 min, the supernatant was retained. The extraction was repeated at least three times with small amounts of acetone until the supernatant remained colorless. The supernatant fractions were pooled and brought to a final volume of 10 mL acetone and filtered through a 0.45 μm × 13 mm PTFE membrane filter (VWR International, Radnor, PA, USA) before analysis.

The quantitative determination of violaxanthin and neoxanthin carotenoids from the wheat plants treated with antibiotics and textile dyes was performed with a liquid chromatograph system (HPLC, Agilent Technologies 1200 Series, Santa Clara, CA, USA) equipped with a diode array detector (DAD). A Zorbax Eclipse XDB-C18 reversed-phase column (4.6 mm i.d. × 150 mm column length, 5 mm particle size, Agilent Technologies), was used and maintained at 10 °C with a flow rate of 1.5 mL min^−1^. The chromatographic elution solutions were buffered ultra-pure water (0.1 M sodium phosphate buffer, pH = 8, A) and HPLC grade acetone (B). The chromatographic elution program is described in Opriş et al. [10].

The elution program used was as follows: first 7.5 min was run isocratically 25% (A) and 75% (B), followed by a 9.5 min linear gradient to 100% (B), which was run isocratically for 3 min. By a 2 min linear-gradient, the eluent composition was changed to 25% (A) and 75% (B) [10].

### 4.4. Separation of Pigment Standards for HPLC Calibration

Due to lack of commercial standards, the reference pigments of interest were obtained by extracting them from plants or algae, followed by chromatographic purification on thin-layer chromatographic (TLC) plates [33]. After separation, TLC plate regions with compounds of interest are removed and extracted in various solvents, and the pigment concentrations are determined by spectrophotometric measurements using reported molar extinction coefficients.

In our study, carotenoid pigments (violaxanthin and neoxanthin) were extracted from fresh leaves of *Primula vulgaris* L., which were powdered in liquid nitrogen with calcium carbonate as wheat samples (both purchased from Sigma-Aldrich, Steinheim, Germany). These two pigments were extracted in 100% ethanol (Sigma-Aldrich) and concentrated in a vacuum evaporator (RV10 digital, IKA^®^-Werke GmbH & Co. KG, Staufen, Germany) and further separated on TLC preparative plates (20 × 20 cm, 500 µm, Analtech, Newark, NJ, USA). The TLC plates were spotted in bands with a Hamilton microsyringe (50 µL, model 705 RN SYR, Hamilton Bonaduz AG, Bonaduz, GR, Switzerland). Further, the TLC plates were developed using a mixed solvent composed of 400 mL petroleum ether (Sigma-Aldrich), 44 mL of 2-propanol (Romil Ltd., Cambridge, UK), and 20 mL ultrapure water [34]. The spotted plates were developed by ascending technique (separation distance 10 cm) for 15 min in a glass chamber previously saturated for 30 min with the mobile phase at 23 °C room temperature. Further, the TLC plates were dried at room temperature, and the extraction of violaxanthin and neoxanthin from the plates was performed in 100% acetone (Sigma-Aldrich). The pigment concentrations were calculated from the spectrophotometric measurements (UV 2550 UV-Vis spectrophotometer, Shimadzu, Kyoto, Japan) using the appropriate wavelengths and extinction coefficients for specific carotenoids.

### 4.5. Statistical Analysis and Data Interpretation

The research was based on three-factor experiments (organic pollutants: antibiotics and textile dyes, the concentration of pollutants used for the plant treatments, and the time exposure of the plants). The experiments were replicated three times with independent samples of plants. All data shown in figures are means of these independent samples ± SEM (standard error of the mean) of each treatment. Means among the treatments were statistically compared with ANOVA followed by post hoc Tukey’s test using the ORIGIN 9.0 program (OriginLab Corporation, Northampton, MA, USA).

In addition, hierarchical cluster analysis (HCA) and principal component analysis (PCA) were used to characterize the coordinated variations in pigment contents across all treatments. Further, PCA was performed with the carotenoid contents from wheat plants to differentiate the treated plants from the control ones. Both HCA and PCA analyses were performed with Minitab 17 (Minitab Ltd., Coventry, UK). All statistical tests were considered significant at *P* < 0.05.

## 5. Conclusions

This study demonstrates how two different types of environmental organic pollutants—antibiotics, and textile dyes—affect two biologically essential carotenoids. Both categories of pollutants negatively influenced the content of violaxanthin and neoxanthin, but the textile dyes constituted more severe stress to the wheat plants. With the increasing concentration of textile dyes, the content of the analyzed carotenoids progressively decreased. In contrast, the overall decreases in antibiotics were smaller, and the dose dependence was less prominent. The present study and other similar studies raise awareness of the impact of diffusely spread environmental pollutants on plants in general and on plants with crucial nutritional value.

## Data Availability

Not applicable.

## Sample Availability

Not available.
